# Robust linear regression model of Ki-67 for mitotic rate in gastrointestinal stromal tumors

**DOI:** 10.3892/ol.2014.1802

**Published:** 2014-01-15

**Authors:** RALF KEMMERLING, DENIS WEYLAND, TOBIAS KIESSLICH, ROMANA ILLIG, ECKHARD KLIESER, TARKAN JÄGER, OTTO DIETZE, DANIEL NEUREITER

**Affiliations:** 1 Institute of Pathology, Paracelsus Medical University/Salzburger Landeskliniken, Salzburg A-5020, Austria; 2 University of Applied Sciences Upper Austria, Bio- and Environmental Engineering, Wels A-4600, Austria; 3 Department of Internal Medicine I, Paracelsus Medical University/Salzburger Landeskliniken, Salzburg A-5020, Austria; 4 Institute of Physiology and Pathophysiology, Paracelsus Medical University, Salzburg A-5020, Austria; 5 Department of Surgery, Paracelsus Medical University/Salzburger Landeskliniken, Salzburg A-5020, Austria

**Keywords:** gastrointestinal stromal tumors, mitosis, proliferation, Ki-67, PHH3

## Abstract

Risk stratification of gastrointestinal stromal tumors (GISTs) by tumor size, lymph node and metastasis status is crucially affected by mitotic activity. To date, no studies have quantitatively compared mitotic activity in hematoxylin and eosin (H&E)-stained tissue sections with immunohistochemical markers, such as phosphohistone H3 (PHH3) and Ki-67. According to the TNM guidelines, the mitotic count on H&E sections and immunohistochemical PHH3-stained slides has been assessed per 50 high-power fields of 154 specimens of clinically documented GIST cases. The Ki-67-associated proliferation rate was evaluated on three digitalized hot spots using image analysis. The H&E-based mitotic rate was found to correlate significantly better with Ki-67-assessed proliferation activity than with PHH3-assessed proliferation activity (r=0.780; P<0.01). A linear regression model (analysis of variance; P<0.001) allowed reliable predictions of the H&E-associated mitoses based on the Ki-67 expression alone. Additionally, the Ki-67-associated proliferation revealed a higher and significant impact on the recurrence and metastasis rate of the GIST cases than by the classical H&E-based mitotic rate. The results of the present study indicated that the mitotic rate may be reliably and time-efficiently estimated by immunohistochemistry of Ki-67 using only three hot spots.

## Introduction

Gastrointestinal stromal tumors (GISTs) are amongst the most common types of non-epithelial tumors of the gastrointestinal tract with an annual incidence of one or two cases per 100,000 individuals. In ~80% of the cases, patient age is between 55 and 65 years with a slight male predominance (60, vs. 40%) (1,2).

Prognostic risk stratification is based on conventional tumor characteristics, such as tumor size, involved lymph nodes and metastases (according to the 7th TNM guidelines) (3). Additionally, the mitotic activity on hematoxylin and eosin (H&E)-stained slides discriminates low and high mitotic GISTs (4). However, it is time-intensive to screen the recommended 50 high-power fields (HPFs) in the pathologist’s daily workflow, and the corresponding area (mm^2^) depends on the microscope setup, which is often not calibrated. The interobserver quality for the detection of mitosis is poor in GISTs and soft tissue sarcoma (5,6), since the mitotic figures are extremely heterogeneous and variably distributed. A useful approach is to detect mitosis with cell cycle markers, such as Ki-67 and phosphohistone H3 (PHH3), which highlight cells in all mitotic phases [without G0 (7) or in the late G2 and M cell cycle phases, respectively (8)]. However, instructions for the evaluation of proliferation based on immunohistochemistry (IHC) currently use heterogeneous cut-off values (9), semi-quantitative scoring of positive cells (10) or counting of 1,000 cells (11,12). Thus, a definitive method of cell counting remains to be established (13) or is unrealistic for the pathologist’s workload. Combining an automated image analysis with quantification tools, standardized instructions may simplify and accelerate the assessment of the mitotic rate in GISTs and other tumors.

Therefore, the current study quantitatively investigated the PHH3- and Ki-67-based mitotic/proliferation activity in GISTs, using IHC and automatic image analysis, to estimate the H&E-based mitosis rate by a linear regression model.

## Materials and methods

### Patient characteristics

The present study included 154 formalin-fixed and paraffin-embedded (FFPE) tissue samples of primary resected GIST between 1997 and 2012 with complete histopathological records (spindle-shaped, epitheloid and mixed morphological types) and classification according to TNM (for details see Table I) (3). The range of fixation time of the obtained specimens was between 12 and 24 h in 4% phosphate-buffered saline solution to avoid false-positive or -negative immunohistochemical staining patterns due to under- or over-fixation (14,15).

### Morphology

Based on conventional 5-μm H&E-stained FFPE sections, mitotic cells were counted in 50 consecutive HPFs according to previous studies (3,4) on a Leica DM2000 microscope (Leica Microsystems, Vienna, Austria) by two independent investigators.

### IHC

IHC was performed using an Autostainer Plus (Dako Österreich GmbH, Vienna, Austria), routinely, according to the manufacturer’s instructions (16). This involved using heat-induced epitope retrieval in pH 9.0 antigen retrieval buffer (Dako Österreich GmbH) at 95ºC for 40 min for the Ki-67 (mouse monoclonal; 1:500; Dako Österreich GmbH) and PHH3 (rabbit polyclonal; 1:200; Cell Marque Corporation, Rocklin, CA, USA) antibodies.

### Interpretation of IHC

PHH3-positive cells were counted manually in 50 HPFs. The Ki-67-based proliferation rate was assessed by the optimized particle analysis module according to the software manual (ImageAccess 9 Enterprise; Imagic Bildverarbeitung AG, Glattbrugg, Switzerland) on three digitized hot spot areas and associated with 50 HPFs per mm^2^ tissue section area, as well as the total number of cells.

### Ethics

The present study was conducted following our national and institutional guidelines, as well as in accordance with the Declaration of Helsinki (1964). Based on the retrospective nature of this study and full anonymization of the patient data, the current study was not subject to formal approval of the appropriate local ethics comitee.

### Statistical analysis

Statistical analysis was performed using IBM^®^ SPSS^®^ 20.0 (IBM Corporation, New York, NY, USA). Kendall’s rank two-tailed test, Spearman’s rank correlation test and a linear regression analysis were used for correlation analysis and for the development of a prognostic model for the mitosis rate on H&E sections. The distribution of mitosis and proliferation rate was analyzed by Kolmogorov-Smirnov test as well as by Monte Carlo sequence analysis. The interobserver agreement for mitosis rate on H&E sections was calculated using the κ-statistic. The Wilcoxon signed-rank test/Student’s t-test and univariate analysis of variance (ANOVA) were applied for differences between two or more groups of tissue samples, respectively. For survival analysis, cases with a missing date of mortality were excluded. Univariate survival analysis was performed by the Kaplan-Meier method comparing the survival curves with the log-rank test. P<0.05 was considered to indicate a statistically significant difference.

## Results

### Patient characteristics and their association with H&E-based mitosis rate and PHH3-/Ki-67-based mitotic/proliferation count

According to Table I, the majority of the 154 GISTs were of gastric origin (n=96; 62.3%) with a mean tumor size of 4.90±3.81 cm [mostly pT2 according to TNM (3)], and predominantly with spindle-shaped cell pattern (n=92; 59.7%), low mitotic activity (4) and affecting more females (n=91; 59.1%) than males.

Assessment of the mitotic and proliferation rate revealed a significant increase between the observed H&E-stained mitotic rate and the PHH3-based mitotic rate and, particularly, the Ki-67-based proliferation rate (P<0.001), whereby the calculated range was relatively high as reflected by the standard deviation (Table I). Overall, no normal distribution of mitotic and proliferation rate was observed (Kolmogorov-Smirnov test). Additionally, the H&E mitosis rate revealed a random distribution (for ~96% of the GIST cases; Monte Carlo sequence analysis). The interobserver agreement for detection of H&E mitosis was moderate (κ=0.562).

The mitotic/proliferation rate differed significantly between spindle-shaped and epithelioid morphologies (ANOVA; P<0.05). Furthermore, comparison of the clinical and morphological results revealed a significant difference in H&E-based mitosis and Ki-67-based proliferation rates (ANOVA; P<0.05) between T1 and T3 stages, whereas no differences in mitosis and proliferation were observed with regard to primary tumor localizations.

### Correlation analysis of mitotic and proliferation rate

Correlation analysis highlighted a significant correlation between H&E-based mitosis rate and PHH3- (Pearson’s product moment correlation coefficient; r=0.457; P<0.01) or Ki-67-based proliferation status per 50 HPFs or mm^2^, respectively (r=0.780; P<0.01).

### Linear regression model for H&E mitotic rate

**Figure 1:**
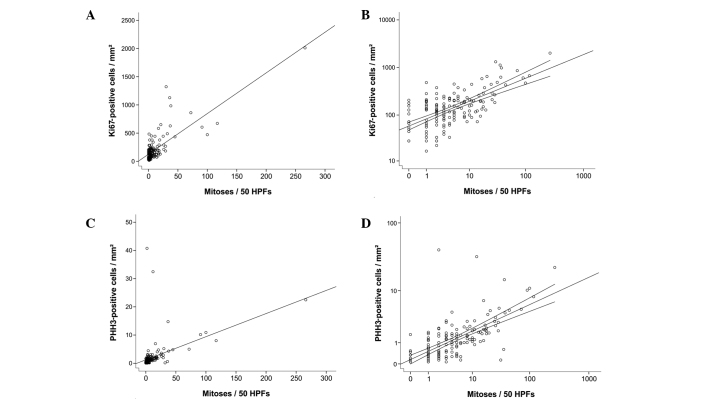
Correlation between PHH3/Ki-67 and H&E mitosis rates. (A and C, linear; B and D, logarithmic scales) Scatter plots with regression lines demonstrating the correlation between PHH3-based mitotic activity and Ki-67-based proliferation activity and conventional H&E-based mitotic rate per 50 HPFs indicating the following: (i) Differences between the two immunohistochemistry markers; and (ii) variances in the two markers compared with H&E-associated mitotic rate, particularly in the low mitotic ranges (in B and D the lines indicate the confidence interval of the mean). PHH3, phosphohistone H3; H&E, hematoxylin-eosin; HPFs, high power fields.

Linear regression analysis reached high significance levels (ANOVA; P<0.001) with a combination of the two IHC markers or Ki-67 alone, whereas the highest significance levels for the intercept term and slope value of the linear regression were reached using the Ki-67 expression analysis per mm^2^ using the following equation: f(x) = 0.084x – 6.328 (Table II and Fig. 1). Using the published cut-off of 5 per 50 HPFs, discriminating GISTs with low (<5 per HPF) and high (>5 per HPF) mitotic rates (4) as variables, the equation was re-calculated as follows: 5=0.084x – 6.328, where x= 134.8 per mm^2^. Therefore, GISTs with low or high proliferation rates may be classified with a threshold value of 134.8 Ki-67-positive cells per mm^2^ and this mathematical model may be used for the rapid calculation of the H&E-based mitotic rate.

### Correlation between recurrence, metastases and survival, and mitotic and proliferation rates

**Figure 2:**
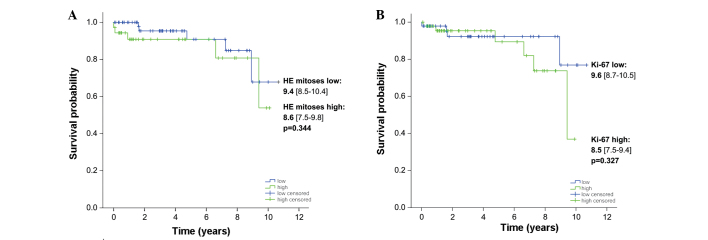
Kaplan-Meier survival analysis was performed for GIST cases with (A) low and high mitotic rates of conventional H&E-based assessment according the TNM (3,4) and compared with (B) low and high proliferation rates of Ki-67 immunohistochemistry using the threshold value of 134.8 Ki-67-positive cells per mm^2^, based on the described linear regression model. The survival analysis indicated that none of the applied methodological strategies exhibited an improved or significant prognostic value on the survival of patients with GIST [Mantel-Cox (log-rank) test; H&E vs. Ki-67, P=0.344 vs. 0.327]. H&E, hematoxylin-eosin; GIST, gastrointestinal stromal tumors.

The statistical analysis of the rate of recurrence and metastases indicated that Ki-67 exhibits a higher prognostic impact on the recurrence and metastases of GISTs compared with pHH3 or H&E (P<0.01; Table III). Although the Kaplan-Meier survival analysis revealed no significant difference in the survival rate of patients with GIST using the classical cut-off of 5 H&E mitoses per 50 HPFs or the threshold value of 134.8 Ki-67-positive cells per mm^2^ (Fig. 2), the statistical analysis showed by trend an improved prognosis based on the image analysis of only three hot spots of Ki-67 IHC in Ki-67-low cases.

## Discussion

The quantitative investigation of mitotic and proliferative activity in 154 GISTs revealed the following: i) H&E-based mitotic activity correlates better with Ki-67-based proliferation than with PHH3-based mitotic status; and ii) we provided a mathematical model for H&E-associated mitotic rate assessment based on Ki-67 IHC on three hot spots per mm^2^.

As consensually discussed (4), the prognosis of GIST depends on location, size and mitotic activity. For mitotic activity particularly, it is currently recommended to analyze 50 independent HPFs, which is a rather time-consuming approach. The interobserver κ-values of mitosis are poor for GIST (as demonstrated in the present study) or other soft tissue sarcomas, and even lower for other markers of mitotic/proliferative activity (5,6). This is possibly caused by a non-standardized definition of mitosis, as previously suggested by Miettinen and Lasota (13). As mitoses on H&E staining are randomly distributed throughout the 50 HPFs in >95% of GIST cases, an investigation of only 10–20 HPFs is likely to result in considerable sampling errors. Additionally, HPF size depends on the microscope used and is not usually described in the diagnosis report.

As PHH3 and Ki-67 marked higher proportions of mitotic cells, the investigation of 50 HPFs in 154 GIST cases (i.e. 7,700 HPFs) yielded a calculating prediction model for H&E-based mitotic rate by PHH3 and/or Ki-67. The present study defined a cut-off value of 134.8 Ki-67-positive cells per mm^2^ for discriminating low versus high proliferative GISTs, according to the TNM classification. Currently, no standards for the quantification of Ki-67 have been accepted or recommended (13), as available published data describe only semi-quantitative (9,10) or impossible approaches (11,12). Notably, Ki-67 exhibits an improved prognostic value compared with PHH3 on the rate of recurrence and metastasis of GIST. This supports the impact of the quantitative assessment of Ki-67-associated proliferation as shown in other tumor entities, such as breast cancer or malignant melanoma (17,18).

Nevertheless, the recommended method must be validated by further studies prior to replacing the classical histological study of the mitotic index in GIST. Using the formula developed in the current study, a (semi-) automatic imaging and image analysis system is likely to provide an alternative and more rapid and reliable (based on three IHC hot spots) assessment of the mitosis rate in GISTs compared with the time-consuming H&E-based approach.

## Figures and Tables

**Table I:** Clinical characteristics of GIST cases and distribution of mitosis in H&E-stained specimens and PHH3-/Ki-67-based mitotic/proliferation rates.

Characteristics	GISTs	Esophageal	Gastric	Small intestinal	Rectal
n (%)	154 (100)	1 (0.6)	96 (62.3)	54 (35.1)	3 (1.9)
Female	91 (59.1)	-	55 (57.3)	34 (63.0)	2 (66.7)
Male	63 (40.9)	1 (100.0)	41 (42.7)	20 (37.0)	1 (33.3)
Age, years (mean ± SD)	66.6±14.8	64.1^c^	67.6±11.9	64.1±16.6	59.1±7.2
Female	65.6±14.8	-	67.6±13.0	62.8±17.2	57.7±9.5
Male	67.1±12.1	64.1^c^	67.7±10.5	66.3±15.5	62^c^
Growth pattern^a^, s/e/m	92/31/31	1/0/0	53/22/21	36/8/10	2/1/0
Size, cm (mean ± SD)	4.9±3.8	3.0^c^	4.9±4.0	4.9±3.4	2.9±2.1
T staging^b^, 1-2-3-4	30-81-29-14	0-1-0-0	18-51-19-8	11-27-10-6	1-2-0-0
Mitotic activity, low/high^b^	99/55	1/0	62/34	34/20	2/1
H&E mitotic rate, % (mean ± SD per 50 HPFs, per mm^2^)	10.8±26.0	1^c^	9.0±27.4	14.8±25.7	3.0±2.6
	0.71±1.73	0.06^c^	0.58±1.79	0.96±1.68	0.19±0.17
PHH3-associated mitotic rate, % (mean ± SD per 50 HPFs, per mm^2^)	31±70	7^c^	31±83	32±44	8±6
	10±23	2^c^	10±27	10±14	2±2
Ki-67-associated proliferation rate^d^, % (mean ± SD per 50 HPFs, per mm^2^)	612±737	133^c^	548±699	736±810	600±517
	204±245	44^c^	182±233	245±270	200±172
	2.16±3.88	0.67^c^	1.89±3.56	2.78±4.47	0.05±0.04

a s, spindle cell; e, epitheloid; and m, mixed type;

b according to the current TNM guidelines [7th edition, 2010 (3,4)];

c only one case, unable to calculate due to the low number of cases;

d associated with the number of cells per three HPFs (magnification, ×400), counted by the particle analysis module (ImageAccess 9 Enterprise).

GIST, gastrointestinal stromal tumor; H&E, hematoxylin-eosin, HPFs, high-power fields; PHH3, phosphohistone H3; SD, standard deviation; T, tumor.

**Table II:** Overview of applied linear regression models for H&E mitotic rate.

	ANOVA			Intercept		Slope	
				
Variable	F	P-value	R	Coefficient	P-value	Coefficient	P-value
PHH3/50 HPFs	40.1	<0.001	0.457	5.5	0.009	0.171	<0.001
PHH3/mm^2^	40.1	<0.001	0.457	5.5	0.009	2.547	<0.001
Ki-67, %	46.1	<0.001	0.482	3.7	0.086	3.301	<0.001
Ki-67^a^/mm^2^	235.6	<0.001	0.780	−6.328	<0.001	0.084	<0.001

a Analysis of Ki-67-based proliferation per 50 HPFs revealed similar statistical results.

H&E, hematoxylin-eosin; ANOVA, analysis of variance; PHH3, phosphohistone H3; HPFs, high-power fields.

**Table III:** Correlation between recurrence, metastases and survival, and mitotic and proliferation rates.

Variable	n (%)	H&E mitotic rate (mean ± SD per 50 HPFs)	PHH3-based mitotic rate (mean ± SD per mm^2^)	Ki-67-based proliferation rate (mean ± SD per mm^2^)
Recurrence				
Yes	14 (9.1)	14.6±15.1	2.8±3.8	461.5±418.7^c^
No	140 (90.9)	10.5±27.4	2.0±4.8	178.3±206.9^c^
Metastases				
Yes	14 (9.1)	42.1±73.5^b^	4.3±6.0^b^	469.4±557.0^c^
No	140 (90.9)	7.7±12.8^b^	1.8±4.5^b^	177.5±172.5^c^
Survival^a^				
Yes	10 (6.5)	8.6±15.3	1.9±4.5	187.4±186.4
No	144 (93.5)	42.9±84.1	4.0±7.2	444.4±638.4

a Survival indicates whether patient was alive at the time point of investigations.

b P<0.05 and

c P<0.01, indicating significant differences within each category.

H&E, hematoxylin-eosin; HPFs, high-power fields; PHH3, phosphohistone H3.

## Abbreviations

GIST: gastrointestinal stromal tumor
H&E: hematoxylin-eosin
HPF: high power field
